# Monitoring of ant species surrounding the ports of South Korea

**DOI:** 10.3897/BDJ.13.e142634

**Published:** 2025-01-20

**Authors:** Dayeong Kim, Heejo Lee, Nanghee Kim, Beom-jun Jang, Dong Eon Kim

**Affiliations:** 1 Invasive Alien Species Team, National Institute of Ecology, Seocheon 33657, Republic of Korea Invasive Alien Species Team, National Institute of Ecology Seocheon 33657 Republic of Korea; 2 National Ecosystem Survey Team, National Institute of Ecology, Seocheon 33657, Republic of Korea National Ecosystem Survey Team, National Institute of Ecology Seocheon 33657 Republic of Korea; 3 Environmental Impact Assessment Team, National Institute of Ecology, Seocheon 33657, Republic of Korea Environmental Impact Assessment Team, National Institute of Ecology Seocheon 33657 Republic of Korea; 4 Research Policy Planning Team, National Institute of Ecology, Seocheon 33657, Republic of Korea Research Policy Planning Team, National Institute of Ecology Seocheon 33657 Republic of Korea

**Keywords:** Alien ants, *Solenopsisinvicta*, *Solenopsisgeminata*, *Paratrechinalongicornis*, *Trichomyrmexdestructor*, *Nylanderiabourbonica*, port investigation

## Abstract

The introduction and spread of invasive insects is accelerating worldwide owing to human activities, such as trade and transportation development; in particular, ports are hubs and routes for invasive insects, including ants. We surveyed ant populations in eight ports from 2021 to 2023 using pitfall traps. A total of 316,975 ants belonging to four subfamilies, 26 genera and 44 species were identified as *Tetramoriumtsushimae*, *Lasiusniger*, *Brachyponerachinensis* and *Nylanderiaflavipes*. The statistical analysis showed that the highest values by index were for the Incheon Port (0.25), the diversity index was for the Daesan Port (2.00), the evenness index was for the Daesan Port (0.71) and the richness index was for the Gamman Port (2.13). Non-metric multidimensional scaling analysis (NMDS) indicated that ants around the Ports of Gamman, Ulsan and Gwangyang had more dominant species than those around the other ports. Five species of alien ants, including *Solenopsisinvicta*, *Solenopsisgeminata*, *Paratrechinalongicornis*, *Trichomyrmexdestructor* and *Nylanderiabourbonica* were identified at Gamman Port, *Paratrechinalongicornis* at Ulsan Port and Gwangyang Port. This study provides comprehensive insights into the distribution and status of ants around ports, offering foundational data for the early detection of alien ants to reduce the risk of their settlement and spread and to respond proactively.

## Introduction

The introduction of plants and animals into countries is constantly increasing owing to a combination of factors, including increased trade between countries and international travel due to the development of transportation (Secretariat of the Convention on Biological Diversity 2014). Ants are a ubiquitous and ecologically dominant group of animals. They account for more than half of the world's insect biomass and outnumber all vertebrates in terrestrial ecosystems (Andersen 2021). Ants are a relatively less dispersive species owing to their localised nature; however, they have been introduced worldwide through human trade. The transportation of cargo through ports accounts relatively for 90% of the global trade, making it an important means of introducing alien ants to new areas (Yang et al. 2019), with more than 150 ant species being introduced to new regions (McGlynn 1999, Shingo et al. 2022). Alien ants have the potential to become invasive alien species if they spread globally on ships, trains etc. because of their small size, nesting habitat and opportunistic feeding behaviour (Xu et al. 2022).

Ants are a remarkably invasive species that have been aggressively introduced outside of their native range and have negative impacts on biodiversity, agriculture, ecosystems and human life, with some even acting as vectors of pathogens (McGlynn 1999). Ants survive and reproduce if their new habitat is similar to their native habitat and they spread widely in an area by competing with native species for food and habitats, thereby increasing their populations and damaging ecosystems. Port areas have been reported to be at high risk of invasion by alien ants (Shingo et al. 2022). In particular, *Solenopsisinvicta*, *Solenopsisgeminata* and *Linepithemahumile* have been introduced globally through transportation and are included in the list of the 100 Worst Invasive Alien Species (Andersen 2021).

In Korea, invasive alien ants have been found in and around ports since the red *S.invicta* species was first discovered in Busan Port container yard in 2017 (National Institute of Ecology 2020) and the *L.humile* species was first discovered in the vicinity of Busan Station in 2019, which has been confirmed to date (Lee et al. 2023).

Therefore, as the introduction and settlement of high-risk alien ants are a concern, this study aimed to investigate ants in neighbouring countries, such as China, Japan and Southeast Asia and around ports with high cargo traffic to identify their habitat status and use it as a basis for preventing the spread and settlement of alien ants in the ecosystem during the early stages of their introduction.

## Materials and methods

### Study areas

**Figure 1.**
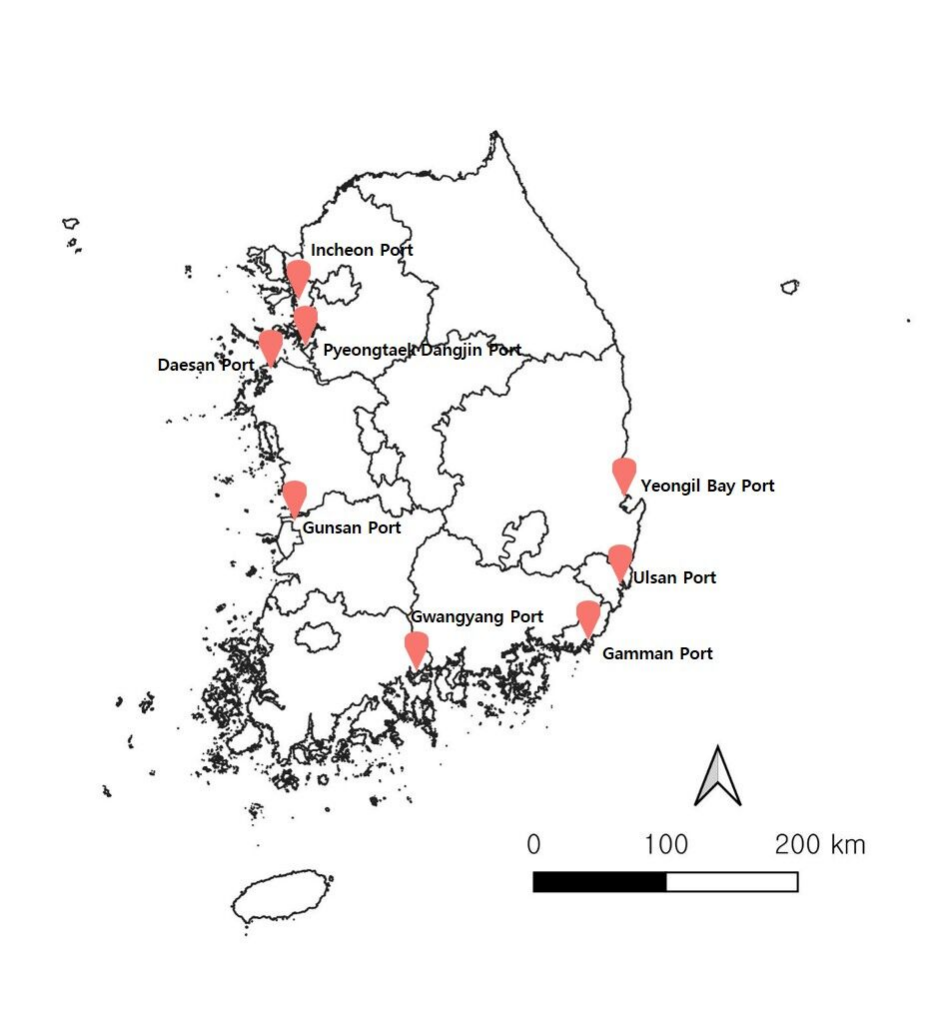
Survey area surrounding the various ports.

Eight ports were selected, based on their total cargo volume (Lee et al. 2021). The areas surrounding the Ports of Gamman, Gunsan, Pyeongtaek Dangjin, Gwangyang, Daesan, Incheon, Ulsan and Yeongil Bay, including grasslands, lawns, forest roads and sunny areas adjacent to container yards, were surveyed once a month from April to November over the course of 3 years (2021–2023) (Fig. 1).

### Investigation methods

**Figure 2.**
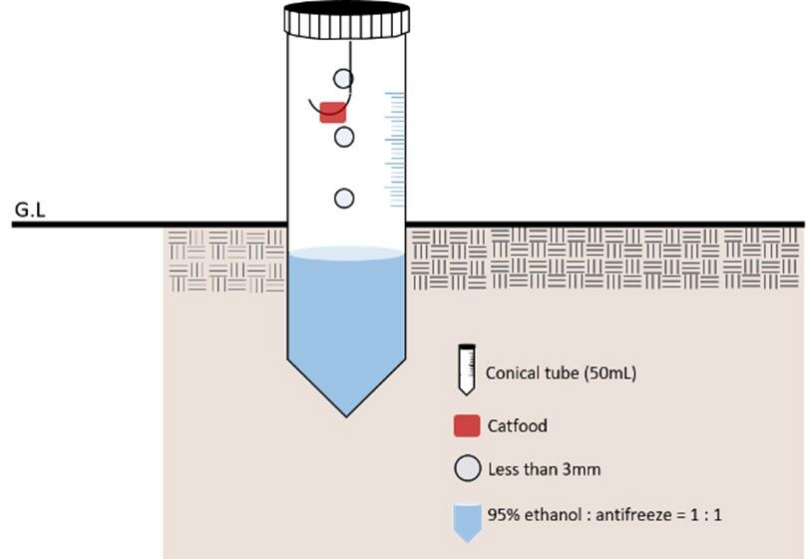
Pitfall trap schematic.

The ant survey was conducted between 10:00 h and 16:00 h on a day with favourable weather conditions. We placed 50 pitfall traps per port in the surrounding grassy area within a 50-m radius of the port, spaced 30 to 50 m apart. The pitfall traps consisted of a 50 ml conical tube with a hole no larger than 3 mm in diameter. For long-term preservation inside the conical tubes, we made a 1:1 mix of eco-friendly conservation solution (ethylene glycol) and ethanol (ethyl alcohol) and used attractants (cat-mini-real chicken and salmon cubes) as insect traps. Ants collected from the pitfall traps were re-installed 3–4 weeks after installation (Fig. 2).

The collected specimens were classified and identified under a stereomicroscope (OLYMPUS SZX16) and camera (OLYMPUS, DP27) in the laboratory of the National Institute of Ecology and stored by liquid immersion (95% ethanol).

### Statistics analysis

We calculated the dominance index (DI: McNaughton 1967), diversity index (H': Pielou 1966), richness index (RI: Margalef 1973) and evenness index (EI: Pielou 1975) for the ants documented in each port and year using the equations below:

- Dominance index = (n1+n2)/N
- Diversity index = \begin{varwidth}{50in}\begin{equation*}
            -∑(i=1)^s[ni/N ln ni/N] 
        \end{equation*}\end{varwidth}
- Richness index = (S-1)/ln(N)
- Evenness index = Hʹ/ln(S)

(n1 is the number of dominant species, n2 is the number of subdominant species, N is the total number of individuals, ni is the number of species i and S is the total number of species).

To analyse the species composition of ants according to region, a data sheet was created, based on the number of ants collected per port per year and calculated using the dissimilarity method (Bray–Curtis) (Bray and Curtis 1957). Based on the dissimilarity matrix, analysis was performed using non-metric multidimensional scaling (NMDS). Correlation analysis of the NMDS results was conducted using a two-dimensional plot of sites and populations within the R environment (Team 2021) and the correlation coefficients and significance levels were calculated using the envfit function in the Vegan package (Oksanen et al. 2022).

## Results

### Total occurrences and dominant species in the port area

A total of 316,975 ants belonging to four subfamilies, 26 genera and 44 species were identified in the areas surrounding the ports (Table 1). The most abundant species was *Tetramoriumtsushimae* (217,900 individuals), followed by *Lasiusniger* (19,977 individuals), *Brachyponerachinensis* (19,011 individuals), *Nylanderiaflavipes* (12,242 individuals), *Lasiushayashi* (12,061 individuals) and *Crematogastermatsumurai* (6,483 individuals).

Seven species were found in all ports; *Lasiusniger*, *Nylanderiaflavipes*, *Crematogastermatsumurai*, *Pristomyrmexpunctatus*, *Solenopsisjaponica*, *Tetramoriumtsushimae* and *Brachyponerachinensis*.

In 2021, a total of 72,550 ants belonging to four subfamilies, 22 genera and 32 species were identified, with the most abundant being *T.tsushimae* (56,515 individuals), *N.flavipes* (3,294 individuals) and *L.niger* (2,424 individuals).

In 2022, a total of 84,805 ants belonging to four subfamilies, 22 genera and 35 species were identified, with the most abundant being *T.tsushimae* (62,869 individuals), *L.niger* (5,409 individuals) and *N.flavipes* (3,976 individuals).

In 2023, a total of 159,620 ants belonging to four subfamilies, 20 genera and 30 species were identified, with the most abundant being *T.tsushimae* (98,516 individuals), *B.chinensis* (16,948 individuals) and *L.niger* (12,144 individuals).

### Appearance and dominant species according to port

**Figure 3.**
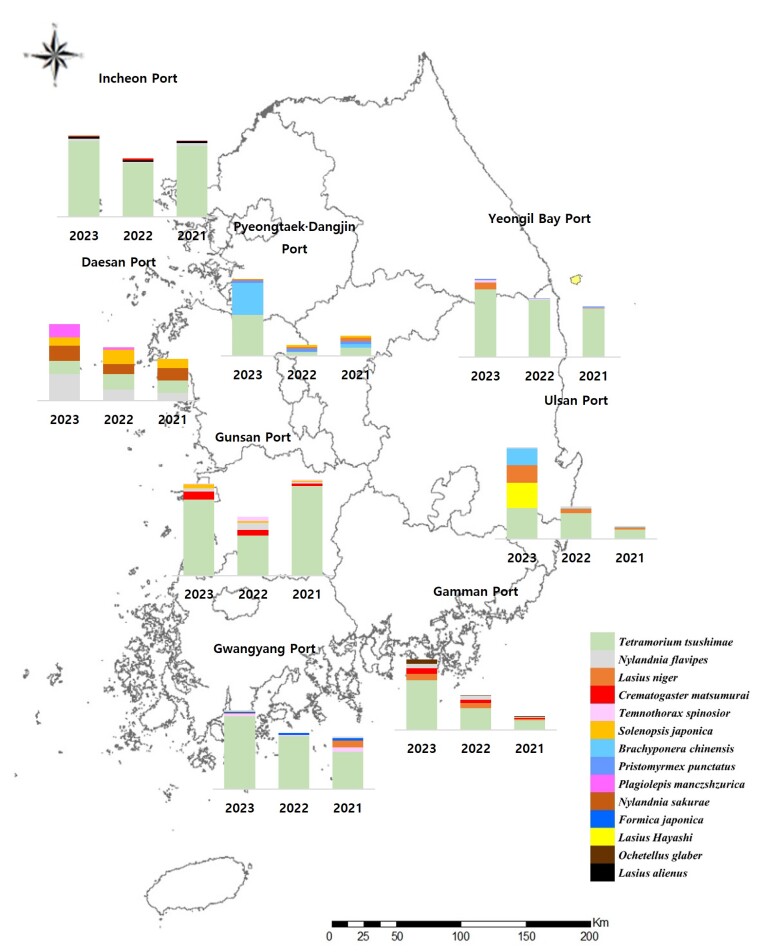
Dominant ant species identified in traps according to different ports.

Based on the port, the following individuals were collected in the pitfall traps (Fig. 3).

In the Gamman Port, a total of 38,513 ants belonging to four subfamilies, 19 genera and 28 species were identified, of them: *T.tsushimae* were 22,978 (14,148 (62.4%) in 2023 / 6,108 (51.9%) in 2022 / 2,722 (67.2%) in 2021), *L.niger* were 3,616 (1,748 (7.7%) in 2023 / 1,509 (12.8%) in 2022 / 359 (8.9%) in 2021), *Crematogastermatsumurai* were 2,747 (1,588 (7.0%) in 2023 / 883 (7.5%) in 2022 / 276 (6.8%) in 2021) and *N.flavipes* were 2,743 (1,248 (5.5%) in 2023 / 1,228 (10.4%) in 2022 / 267 (6.6%) in 2021).

In the Gunsan Port, a total of 9,681 ants belonging to four subfamilies, 13 genera and 14 species were collected, of them: *T.tsushimae* were 7,805 (2,888 (79.3%) in 2023 / 1,515 (64.9%) in 2022 / 3,402 (91.8%) in 2021), *C.matsumurai* were 587 (291 (8.0%) in 2023 / 211 (9.0%) in 2022 / 85 (2.3%) in 2021), *N.flavipes* were 463 (132 (3.6%) in 2023 / 273 (11.7%) in 2022 / 58 (1.6%) in 2021) and *Solenopsisjaponica* were 271 (146 (4.0%) in 2023 / 71 (3.0%) in 2022 / 54 (1.5%) in 2021).

In the Pyeongtaek Dangjin Port, a total of 32,029 ants belonging to four subfamilies, 15 genera and 21 species were collected, of them: *T.tsushimae* were 13,728 (10,591 (51.3%) in 2023 / 957 (21.6%) in 2022 / 2,180 (31.4%) in 2021), *B.chinensis* were 9,290 (8,265 (40.0%) in 2023 / 166 (3.7%) in 2022 / 859 (12.4%) in 2021), *Pristomyrmexpunctatus* were 2,111 (569 (2.8%) in 2023 / 830 (18.7%) in 2022 / 712 (10.3%) in 2021) and *L.niger* were 1,680 (324 (1.6%) in 2023 / 411 (9.3%) in 2022 / 945 (13.6%) in 2021).

In the Gwangyang Port, a total of 38,917 ants belonging to four subfamilies, 15 genera and 19 species were collected, of them: *T.tsushimae* were 30,944 (13,843 (85.1%) in 2023 / 10,034 (76.8%) in 2022 / 7,067 (73.8%) in 2021), *Temnothoraxspinosior* were 1,598 (467 (2.9%) in 2023 / 256 (2.0%) in 2022 / 875 (9.1%) in 2021), *L.niger* were 1,361 (86 (0.5%) in 2023 / 0 (0.0%) in 2022 / 1,275 (13.3%) in 2021) and *Formicajaponica* were 982 (239 (1.5%) in 2023 / 310 (2.4%) in 2022 / 433 (4.5%) in 2021).

In the Daesan Port, a total of 11,431 ants belonging to four subfamilies, 14 genera 17 species were collected, of them: *N.flavipes* were 2,569 (1,502 (27.3%) in 2023 / 624 (18.3%) in 2022 / 443 (17.5%) in 2021), *T.tsushimae* were 2,343 (757 (13.8%) in 2023 / 880 (25.8%) in 2022 / 706 (27.9%) in 2021), *N.sakurae* were 2,131 (853 (15.5%) in 2023 / 577 (16.9%) in 2022 / 701 (27.7%) in 2021) and *S.japonica* were 1,808 (493 (9.0%) in 2023 / 803 (23.6%) in 2022 / 512 (20.2%) in 2021).

In the Incheon Port, a total of 82,605 ants belonging to four subfamilies, 12 genera and 14 species were collected, of them: *T.tsushimae* were 75,928 (28,753 (92.6%) in 2023 / 20,335 (90.3%) in 2022 / 26,840 (92.4%) in 2021), *N.flavipes* were 2,375 (8366 (2.7%) in 2023 / 502 (2.2%) / 1,037 (3.6%) in 2021), *L.alienus* were 2,214 (842 (2.7%) in 2023 / 589 (2.6%) in 2022 / 783 (2.7%) in 2021) and *C.matsumurai* were 853 (122 (0.4%) in 2023 / 632 (2.8%) in 2022 / 99 (0.3%) in 2021).

In the Ulsan Port, a total of 67,953 ants belonging to four subfamilies, 14 genera and 19 species were collected, of them: *T.tsushimae* were 31,176 (14,665 (32.7%) in 2023 / 12,148 (75.6%) in 2022 / 4,363 (64.5%) in 2021), *L.Hayashi* were 11,933 (11,933 (26.6%) in 2023 / 0 (0.0%) in 2022/ 0 (0.0%) in 2021), *L.niger* were 11,407 (8,272 (18.4%) in 2023 / 2,103 (13.1%) in 2022 / 1,032 (15.3%) in 2021) and *B.chinensis* were 8,482 (7,992 (17.8%) in 2023 / 301 (1.9%) in 2022 / 189 (2.8%) in 2021).

In the Yeongil Bay Port, a total of 35,846 ants belonging to three subfamilies, 11 genera and 13 species were collected, of them: *T.tsushimae* were 35,846 (12,871 (86.1%) in 2023 / 10,892 (97.3%) in 2022 / 9,235 (95.2%) in 2021), *L.niger* were 1,366 (1,271 (8.5%) in 2023 / 1,271 (8.5%) in 2022 / 7 (0.1%) in 2022 / 88 (0.9%) in 2021), 725 were *T.spinosior* (496 (3.3%) in 2023, 152 (1.4%) in 2022 and 77 (0.8%) in 2021) and 438 were *P.punctatus* (169 (1.1%) in 2023, 67 (0.6%) in 2022 and 202 (2.1%) in 2021).

**Figure 4.**
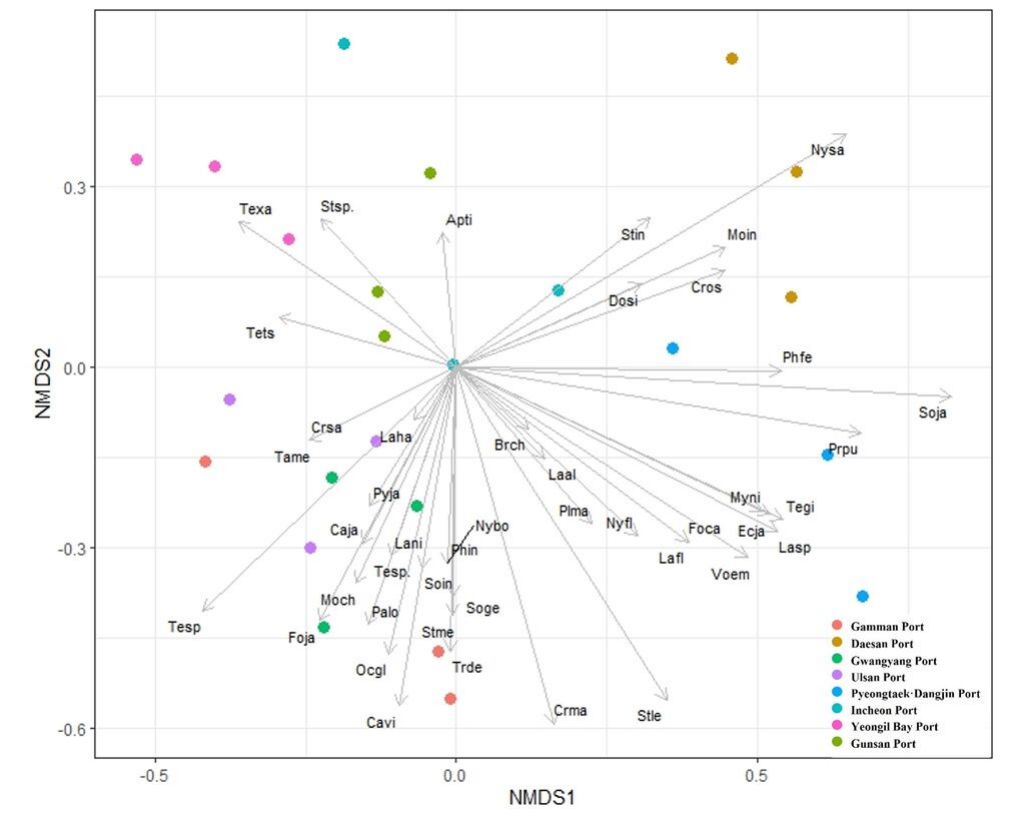
Relationship between ant species and abundance collected per port visualised by non-metric multidimensional scaling (NMDS) analysis. The direction and length of the arrows indicate the correlation between ant species collected at a port. Abbreviations Apti: *Aphaenogastertipuna*, Brch: *Brachyponerachinensis*, Caja: *Camponotusjaponicus*, Cavi: *Camponotusvitiosus*, Crma: *Crematogastermatsumurai*, Cros: *Crematogasterosakensis*, Crsa: *Cryptoponesauteri*, Dosi: *Dolichoderussibiricus*, Ecja: *Ectomomyrmexjavanus*, Foca: *Formicacandida*, Foja: *Formicajaponica*, Laal: *Lasiusalienus*, Lafl: *Lasiusflavus*, Laha: *Lasiushayashi*, Lani: *Lasiusjaponicus*, Lasp: *Lasiusspathepus*, Moch: *Monomoriumchinense*, Moin: *Monomoriumintrudens*, Myni: *Myrmecinanipponica*, Nybo: *Nylanderiabourbonica*, Nyfl: *Nylanderiaflavipes*, Nysa: *Nylanderiasakurae*, Ocgl: *Ochetellusglaber*, Palo: *Paratrechinalongicornis*, Phfe: *Pheidolefervida*, Phin: *Pheidoleindica*, Plma: *Plagiolepismanczshurica*, Prpu: *Pristomyrmexpunctatus*, Pyja: *Pyramicajaponica*, Soge: *Solenopsisgeminata*, Soin: *Solenopsisinvicta*, Soja: *Solenopsisjaponica*, Stin: *Strumigenysincerta*, Stle: *Strumigenyslewisi*, Stme: *Strumigenysmembranifera*, Stsp.: *Strumigenys* sp., Tame: *Tapinomamelanocephalum*, Tegi: *Technomyrmexgibbosus*, Tesp: *Temnothoraxspinosior*, Tesp.: *Temnothorax* sp., Texa:*Temnothoraxxanthos*, Tets: *Tetramoriumtsushimae*, Trde: *Trichomyrmexdestructor*, Voem: *Vollenhoviaemeryi*

NMDS was used to analyse the dominant species in each port (Fig. 4). Longer arrows indicate that the ant species collected at that port was dominant or the only one collected, while shorter arrows indicate that the population was similar to that of other ports. Amongst all of the species collected from the various ports, the dominant species were as follows: *Aphaenogastertipuna* in the Gunsan Port, *Temnothoraxxanthos*, *Strumigenys* sp. in the Yeongil Bay Port, *Strumigenysincerta*, *Nylanderiasakurae*, *Monomoriumintrudens*, *Crematogasterosakensis* in the Daesan Port, *Tapinomamelanocephalum*, *Cryptoponesauteri*, *Pheidoleindica*, *Solenopsisinvicta*, *Nylanderiabourbonica*, *Solenopsisgeminata*, *Strumigenysmembranifera*, *Trichomyrmexdestructor* in the Gamman Port, *Lasiushayashi* and *Pyramicajaponica* in the Ulsan Port, *Camponotusjaponicus* and *Monomoriumchinense* in the Gwangyang Port and *Formicacandida*, *Lasiusspathepus*, *Lasiusflavus*, *Myrmecinanipponica*, *Vollenhoviaemeryi* and *Technomyrmexgibbosus* in the Pyeongtaek Dangjin Port.

### Alien ants species detection status

**Figure 5.**
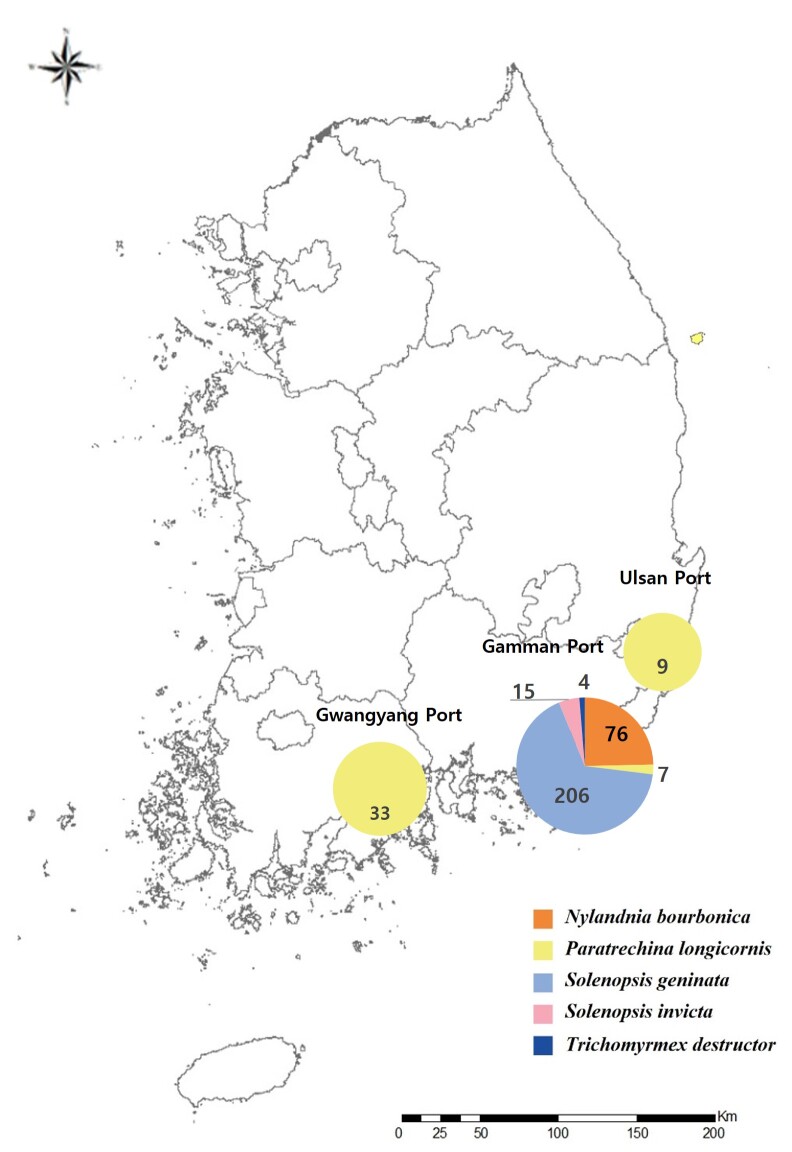
Percentage of alien ants identified in area surrounding ports.

Five species of alien ants were collected from the pitfall traps: *S.invicta*, *S.geminata*, *P.longicornis*, *T.destructor* and *N.bourbonica* (Fig. 5). *S.invicta*, *S.geminate* and *P.longicornis* are designated as ecosystem-disturbing species by the Ministry of Environment and controlled pests by the Ministry of Agriculture, Forestry and Fisheries, *T.destructor* is designated as a controlled pest by the Ministry of Agriculture, Forestry and Fisheries (Ministry of Agriculture, Food and Rural Affairs and Quarantine 2024, Ministry of Environment 2024).

**Figure 6.**
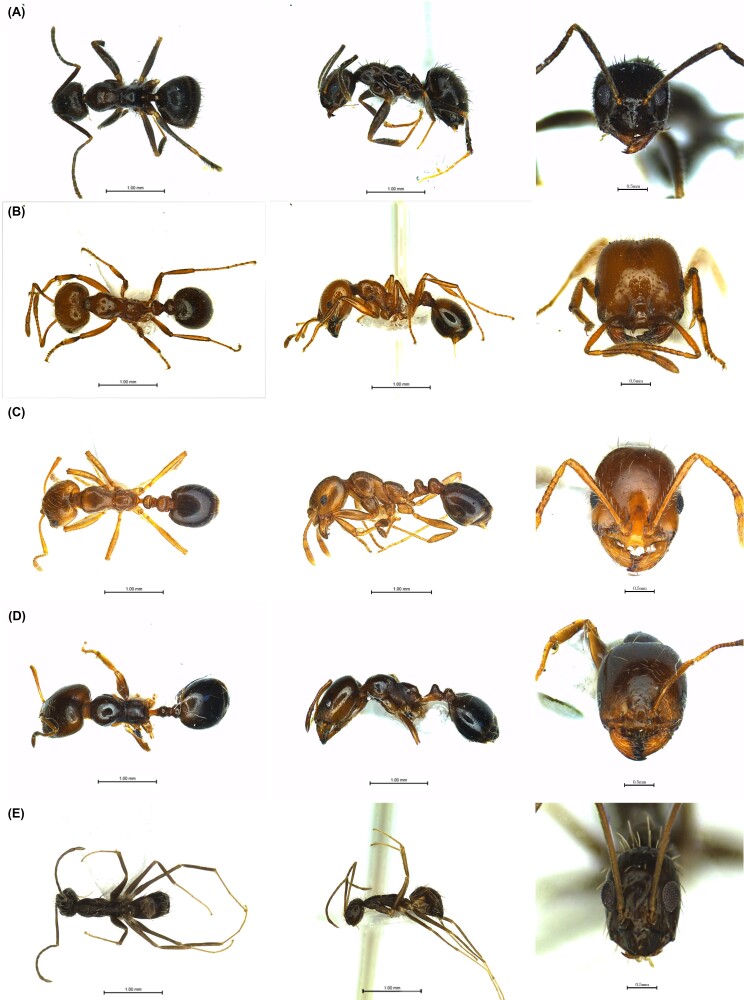
Photographs of alien ant specimens collected from surrounding port areas. Dorsal, lateral and frontal view of **A** *Nylanderiabourbonica*; **B** *Solenopsisgeminata*; **C** *Solenopsisinvicta*; **D** *Trichomyrmexdestructor*; **E** *Paratrechinalongicornis*.

In 2022, 206 individuals of *S.geminata* (Fig. 6B), seven individuals of *P.longicornis* (Fig. 6E) and three individuals of *T.destructor* (Fig. 6D) were collected from the area around the Gamman Port, 33 individuals from the area around Gwangyang Port and nine individuals from the area around Ulsan Port of *P.longicornis* were collected. In 2023, 15 individuals of *S.invicta* (Fig. 6C), 76 individuals of *N.bourbonica* (Fig. 6A) and one individuals of *T.destructor* were collected from the area around Gamman Port. Alien ants were collected in three out of eight peripheral port areas at a rate of 11.36% (Fig. 6).

### Statistics analysis according to port

**Statistics analysis organised by port.**

The dominance index (DI) in the areas around eight ports was the lowest in the Daesan Port (0.02) and the highest in the Incheon Port (0.25), but there was no significant difference between the ports and the DI was relatively low. Ants of 14 species were collected from the Incheon Port, which was fewer than the 17 species collected from the Daesan port; however, the DI was higher. The diversity index (H') was the lowest at the Yeongil Bay Port (0.39) and the highest at the Daesan Port (2.0), but overall, the diversity within the colonies seemed to be low. The evenness index (EI) was the lowest for the Yeongil Bay Port (0.15) and the highest for the Daesan Port (0.71); however, the overall index was low, indicating that the biota was not evenly distributed. The richness index (RI) was the lowest at the Yeongil Bay Port (0.95) and the highest at the Gamman Port (2.13), but the overall values were low, suggesting that biodiversity was not high (Table 2).

**Statistics analysis of areas surrounding ports organised by year**

In 2021, the DI was the lowest for the Daesan Port (0.02) and the highest for the Incheon port (0.38); however, overall, it was relatively low for each port. The H' was the lowest for the Yeongil Bay Port (0.26) and the highest for the Pyeongtaek Dangjin Port (2.05). The EI was the lowest for the Yeongil Bay Port (0.13) and the highest for the Daesan Port (0.73). The RI was the lowest for the Incheon Port and the Yeongil Bay Port (0.63) and the highest for the Pyeongtaek Dangjin Port (1.52).

In 2022, the DI was the lowest for the Ports of Gunsan, Pyeongtaek Dangjin and Daesan (0.02) and the highest for the Incheon Port (0.25). The H' was the lowest for the Yeongil Bay Port (0.16) and the highest for the Pyeongtaek Dangjin Port (2.19). The EI was the lowest for the Yeongil Bay Port (0.07) and the highest for the Pyeongtaek Dangjin Port (0.76). The RI was the lowest for the Yeongil Bay Port (0.88) and the highest at the Gamman Port (1.76).

In 2023, the DI was the lowest at the Daesan Port (0.01) and the highest at the Incheon Port (0.19). The H' was the lowest in the Incheon Port (0.37) and the highest in the Daesan Port (2.07). The EI was the lowest at the Incheon Port (0.15) and the highest at the Daesan Port (0.75). The RI was the lowest for the Yeongil Bay Port (0.75) and the highest for the Gamman Port (1.67) (Table 2).

## Discussion

In this study, a total of 316,975 individuals ants belonging to four subfamilies, 26 genera and 44 species and 350 individuals alien ants belonging to two subfamilies, four genera and five species were identified. The number of ant species in the areas surrounding each port varied somewhat, ranging from 13 to 28 species, which was believed to be due to differences in the environment and location conditions of the collection areas. At the Yeongil Bay Port, the surrounding area has a concrete floor, whereas in the Gamman Port, it is composed of grassland and scrub forests, which provide an easy food supply and suitable habitat for nesting, which may explain the diverse species composition.

*Tetramoriumtsushimae* was the most dominant species in the entire port area, followed by *L.niger*, *B.chinensis*, *N.flavipes* and *L.hayashi*, most of which are widely distributed and densely populated in urban areas and grasslands and have been selected as indicator species for urbanisation (Korea Forest Research Institute 2011). Most of the habitats around the port are areas where trees and scrub forests are found along forest roads and it seems that the ants commonly found in urban centres were dominant and collected.

The DI of the Incheon Port was the highest 0.25, indicating that a single species, *T.tsushimae*, was collected significantly more often than those at other ports. The H’ was 2.00 in the Daesan Port, which is considered a relatively ecologically stable area with a small population, but a wide variety of ant species. The EI was the highest 0.71, indicating that the species were similar in abundance at the other ports. The RI was 2.13 in the Gamman Port, indicating a more balanced species composition and better environmental conditions than the other ports.

The association of ant species collected at the ports through NMDS analysis revealed that the plot locations and arrow directions of the Ports of Gamman, Gwangyang and Ulsan were closely aligned, indicating the presence of similar ant species. In some cases, the species collected in the traps were similar, even though the ports were in different locations; in other cases, the species collected in the traps were different, even though the ports were in similar locations, which may be due to the disturbed environment around the habitat, migration due to foraging activities and the location of the traps. As reported by Li et al. (2023), abiotic variables, such as temperature, precipitation, sunlight and soil characteristics, may also affect the species diversity of the collected ants.

Alien ants are likely to spread and settle throughout the country if they enter the country mixed with containerised cargoes loaded with wood or feed or if they live in containers and are transported (Lee et al. 2008). In a study by Lee et al. (2021), the proportion of containerised cargo was reported to be high in the Ports of Busan, Incheon and Gwangyang. In a study by Sakamoto et al. (2016), ports were reported to serve as entry points for the invasion of other areas. In this study, five species, *S.invicta*, *S.geminata*, *P.longicornis*, *T.destructor* and *N.bourbonica*, were collected from the Gamman Port in Busan and one species of *P.longicornis* was collected from the Ulsan Port and Gwangyang Port, suggesting that they are likely to enter and settle in the surrounding areas of ports along with cargo. Therefore, for ports with a high risk of spreading alien ants to surrounding areas, such as Port of Busan, Ulsan and Gwangyang, it is essential to secure resources for continuous vigilance and prevention as well as invasion response activities. In addition, to prevent the spread of the species inland, it is necessary to regularly monitor container yards to detect alien ants early, fill in gaps in the port floor and remove weeds to create an environment that does not allow ants to live.

Therefore, it is highly likely that ants spread through port transport and are a potential route of introduction. Five species identified in the port areas are likely to settle and spread in South Korea. In other words, it is necessary to establish a regular monitoring plan in the early stages of an invasion by closely inspecting the containerised cargo entering the port, establishing a database of high-risk alien ants likely to be introduced into South Korea and an ongoing monitoring plan for the port area. In addition, an integrated biosecurity system is required to minimise the impact of high-risk alien species introduced into ports. An approach that includes the essential elements of prevention, early detection, rapid response, ongoing management, capacity-building, outreach and research should be considered. In South Korea, it is necessary to establish a response system for high-risk ant species that are likely to be introduced and spread by designating them as management targets between relevant organisations; inspecting border areas and natural ecosystems for the introduction, establishment and spread of these species; and developing measures to block entry routes and effective control, such as monitoring and early warning systems and quarantine of non-plant cargo.

## Figures and Tables

**Table 1.** Ants species identified in the surrounding areas of the ports between 2021 and 2023.

Site, Species	Gamman port	Gunsan port	Pyeongtaek Dangjin port	Gwangyang port	Daesan port	Incheon port	Ulsan port	Yeongil Bay port
**Subfamily Dolichoderinae**								
***Dolichoderus***								
*Dolichoderussibiricus* Emery, 1889					▣ 1	▣ 1		
***Ochetellus***								
*Ochetellusglaber* (Mayr, 1862)	▣ 1,256 ◈ 212 ◎ 202	▣ 1 ◈ 1		▣ 11 ◈ 65 ◎ 84			▣ 352 ◈ 135 ◎ 114	
***Technomyrmex***								
*Technomyrmexgibbosus* Wheeler, 1906			▣ 13 ◈ 6 ◎ 17					
***Tapinoma***								
*Tapinomamelanocephalum* (Fabricius, 1793)	◎ 6							
**Subfamily** **Formicinae**								
***Camponotus***								
*Camponotusvitiosus* Ito, 1912	▣ 59 ◈ 20	▣ 5 ◈ 2 ◎ 1		▣ 6 ◈ 19 ◎ 2			▣ 7 ◈ 4 ◎ 4	
*Camponotusjaponicus* Mayr, 1866	▣14 ◈5			▣ 141 ◈ 178 ◎ 417		▣ 1 ◈ 167	▣ 2 ◈ 1 ◎ 1	▣5
***Formica***								
*Formicacandida* Smith, 1878			◎ 34					
*Formicajaponica* (Motschulsky, 1866)	▣ 308 ◈ 58 ◎ 74		▣ 4	▣ 239 ◈ 310 ◎ 433	▣ 6	▣ 49 ◈ 44 ◎ 197	▣ 53 ◈ 19 ◎ 16	▣ 1 ◈ 1 ◎ 2
***Lasius***								
*Lasiusalienus* (Förster, 1850)	▣ 726 ◈ 581	▣ 1	▣ 65 ◈ 429 ◎ 190	▣ 108		▣ 842 ◈ 589 ◎ 783		
*LasiusHayashi* Yamauchi and Hayashida, 1970					▣ 45 ◈ 58 ◎ 25		▣11,933	
*Lasiusniger* (Linnaeus, 1758)	▣ 1,748 ◈ 1,509 ◎ 359	▣ 6 ◈ 2	▣ 324 ◈ 411 ◎ 945	▣ 86 ◈ 1,275	▣ 192	▣ 245 ◈ 102	▣ 8,272 ◈ 2,103 ◎ 1,032	▣ 1,271 ◈ 7 ◎ 88
*Lasiusspathepus* Wheeler, 1910			◈ 15 ◎ 11					
*Lasiusflavus* (Fabricius, 1781)			◎ 8					
***Nylanderia***								
*Nylanderiabourbonica* (Forel, 1886)	▣ 76							
*Nylanderiaflavipes* (Smith, F., 1874)	▣ 1,248 ◈ 1,228 ◎ 267	▣ 132 ◈ 273 ◎ 58	▣ 186 ◈ 370 ◎ 533	▣ 325 ◈ 149 ◎ 294	▣ 1,502 ◈ 624 ◎ 443	▣ 836 ◈ 502 ◎ 1,037	▣ 665 ◈ 829 ◎ 638	▣ 78 ◈ 1 ◎ 24
*Nylanderiasakurae* (Ito, 1914)	▣ 2		▣ 228 ◈ 92 ◎ 105		▣ 853 ◈ 577 ◎ 701		▣ 15 ◈ 8	
***Paratrechina***								
*Paratrechinalongicornis* (Latreille, 1802)	◈ 7			◈ 33			◈ 9	
***Plagiolepis***								
*Plagiolepismanczshzurica* Ruzsky, 1905	▣ 260 ◈ 165	▣ 88 ◈ 65 ◎ 28	◈ 11 ◎ 4	▣ 194 ◈ 455 ◎ 73	▣ 749 ◈ 159			
**Subfamily Myrmicinae**								
***Crematogaster***								
*Crematogastermatsumurai* Forel, 1900	▣ 1,588 ◈ 883 ◎ 276	▣ 291 ◈ 211 ◎ 85	▣ 52 ◈ 308 ◎ 671	▣ 510 ◈ 109 ◎ 47	▣ 271 ◈ 74 ◎ 98	▣ 122 ◈ 632 ◎ 99	▣ 69 ◈ 52 ◎ 9	▣ 1 ◈ 25
*Crematogasterosakensis* (Forel, 1901)			▣ 3 ◈ 4 ◎ 3		▣ 85 ◈ 10 ◎ 7	▣ 18		
***Monomorium***								
*Monomoriumintrudens* Smith, 1874					▣ 2 ◈ 1			
*Monomoriumchinense* (Santschi, 1925)		▣ 1 ◈ 1		▣ 41 ◈ 49 ◎ 55				
***Myrmecina***								
*Myrmecinanipponica* Wheeler, 1906			◈ 2 ◎ 1					
***Pheidole***								
*Pheidolefervida* Smith, 1874	▣ 1		▣ 214 ◈ 315		▣ 29 ◈ 70 ◎ 21			
*Pheidoleindica* Mayr, 1878	▣ 16							
***Pristomyrmex***								
*Pristomyrmexpunctatus* (Smith, 1860)	▣ 18 ◈ 4 ◎ 17	▣ 4 ◈ 14 ◎ 25	▣ 569 ◈ 830 ◎ 712	▣ 50 ◈ 98 ◎ 72	▣ 459 ◈ 138 ◎ 16	▣ 113 ◈ 50 ◎ 92	▣ 82 ◈ 4	▣ 169 ◈ 67 ◎ 202
***Aphaenogaster***								
*Aphaenogastertipuna* Forel, 1913		◎ 1						
***Pyramica***								
*Pyramicajaponica* Ito, 1914							▣ 1 ◈ 5	
***Solenopsis***								
*Solenopsisjaponica* Wheeler, 1928	▣ 504 ◈ 191 ◎ 3	▣ 146 ◈ 71 ◎ 54	▣ 127 ◈ 519 ◎ 591	▣ 55 ◈ 6 ◎ 36	▣ 493 ◈ 803 ◎ 512	▣ 44 ◈ 19 ◎ 1	▣ 144 ◈ 56 ◎ 71	▣ 20 ◈ 7 ◎ 10
*Solenopsisgeminata* (Fabricius, 1804)	◈ 206							
*Solenopsisinvicta* Buren, 1972	▣ 15							
***Strumigenys***								
*Strumigenysincerta* (Brown, 1949)					◈ 5			
*Strumigenyslewisi* Cameron, 1886	◈ 2		◈ 3 ◎ 6	◈ 2			◈ 3	
*Strumigenysmembranifera* Emery, 1869	▣ 18 ◈ 191							
*Strumigenys* sp.								◈ 1
***Temnothorax***								
*Temnothoraxspinosior* (Forel, 1901)	▣ 296 ◈ 197 ◎ 67	▣ 38 ◈ 155 ◎ 45		▣ 467 ◈ 256 ◎ 875		▣ 1 ◈ 3 ◎ 11	▣ 630 ◈ 396 ◎ 313	▣ 496 ◈ 152 ◎ 77
*Temnothorax* sp.	◈5			▣ 7			◎ 233	◈ 1
*Temnothoraxxanthos* Radchenko, 2004				◎ 3			◎ 15	◎ 58
***Tetramorium***								
*Tetramoriumtsushimae* Emery, 1925	▣ 14,148 ◈ 6,108 ◎ 2,722	▣ 2,888 ◈ 1,515 ◎ 3,402	▣ 10,591 ◈ 957 ◎ 2,180	▣ 13,843 ◈ 10,034 ◎ 7,067	▣ 757 ◈ 880 ◎ 706	▣ 28,753 ◈ 20,335 ◎ 26,840	▣14,665 ◈12,148 ◎4,363	▣ 12,871 ◈ 10,892 ◎ 9,235
***Trichomyrmex***								
*Trichomyrmexdestructor* (Jerdon, 1851)	▣ 1 ◈ 3							
***Vollenhovia***								
*Vollenhoviaemeryi* Wheeler, 1906			◈ 7 ◎ 22					
**Subfamily** **Ponerinae**								
***Brachyponera***								
*Brachyponerachinensis* (Emery, 1895)	▣ 381 ◈ 201 ◎ 57	▣ 42 ◈ 23 ◎ 6	▣ 8,265 ◈ 166 ◎ 859	▣ 188 ◈ 35 ◎ 115	▣ 24 ◈ 7	▣ 12 ◈ 64	▣ 7,992 ◈ 301 ◎ 189	▣ 44 ◈ 40
***Ectomomyrmex***								
*Ectomomyrmexjavanus* Mayr, 1867	◈ 3		◈ 1 ◎ 50		▣ 28	◈ 1		
***Cryptopone***								
*Cryptoponesauteri* (Wheeler, 1906)	◎ 1							
Subfamilies / species	4 / 28	4 / 14	4 / 21	4 / 19	4 / 17	4 / 14	4 / 19	3 / 13
Total	38,513	9,681	32,029	38,917	11,431	82,605	67,953	35,846

Number of individuals by year: ▣: 2023, ◈: 2022, ◎: 2021

**Table 2.** Cluster analysis per year in the port periphery from 2021 to 2023.

Year	Indices*	Gamman port	Gunsan port	Pyeongtaek· Dangjin port	Gwangyang port	Daesan port	Incheon port	Ulsan port	Yeongil Bay port
2021	DI	0.04	0.05	0.04	0.11	0.02	0.38	0.07	0.13
	H'	1.23	0.43	2.05	1.08	1.60	0.36	1.30	0.26
	EI	0.50	0.19	0.71	0.41	0.73	0.18	0.51	0.13
	RI	0.98	0.80	1.52	1.16	0.71	0.63	1.07	0.63
2022	DI	0.09	0.02	0.02	0.13	0.02	0.25	0.17	0.13
	H'	1.71	1.23	2.19	0.99	1.85	0.48	0.90	0.16
	EI	0.56	0.49	0.76	0.36	0.72	0.19	0.32	0.07
	RI	1.76	0.97	1.50	1.32	1.06	0.97	1.32	0.88
2023	DI	0.10	0.02	0.12	0.09	0.01	0.19	0.17	0.09
	H'	1.49	0.86	1.09	0.76	2.07	0.37	1.55	0.56
	EI	0.49	0.33	0.41	0.27	0.75	0.15	0.57	0.24
	RI	1.67	1.00	1.09	1.25	1.25	1.00	1.17	0.75
Total	DI	0.08	0.03	0.07	0.10	0.02	0.25	0.14	0.11
	H'	1.57	0.83	1.66	0.98	2.00	0.41	1.53	0.39
	EI	0.47	0.32	0.55	0.33	0.71	0.16	0.52	0.15
	RI	2.13	1.03	1.58	1.42	1.26	1.03	1.42	0.95

* DI: Dominance index, H’: Diversity index, EI: Evenness index, RI: Richness index.
